# In vitro antioxidant and free-radical scavenging activities of polar leaf extracts of *Vernonia amygdalina*

**DOI:** 10.1186/s12906-023-03923-y

**Published:** 2023-05-04

**Authors:** Endris Muhie Hussen, Sisay Awoke Endalew

**Affiliations:** Department of Chemistry, College of Natural Science, Wollo University, P.O. Box: 1145, Dessie, Ethiopia

**Keywords:** Antioxidants, *Vernonia Amygdalina*, Integrative medicine, Phototherapy, Phosphomolybdenum, Ascorbic acid

## Abstract

**Background:**

Plants are able to deliver a huge number of differing bioactive compounds which may supplement the requirements of the human body by acting as natural antioxidants. Antioxidants are mindful for the defense component of the life form against the pathologies related to the assault of free radicals. The main purpose of this study was to investigate the qualitative phytochemical composition of *Vernonia amygdalina* leaf extract and its antioxidant activity.

**Method:**

The powdered plant sample was successively extracted with aqueous, methanol and ethanol solvents using Soxhlet apparatus. The antioxidant activities of the crude leaf extract were determined using 1, 1- diphenyl-2-picryl hydrazyl (DPPH) radical, 2,2′-azino-bis(3-ethylbenzothiazoline-6-sulfonic acid (ABTS) radical, phosphomolybdate (PM) and hydrogen peroxide (H_2_O_2_) scavenging assay. All the examinations were drained triplicates and average values of each test were taken.

**Results:**

Phytochemical investigation of the plant revealed that the three solvent extracts contained numerous bioactive compounds namely alkaloids, tannins, saponins, phenols, terpenoids, steroids, glycosides and sugars. The result showed that, the leaf extracts of *V. amygdalina* obtained from methanol extract exhibit the maximum antioxidant activity compared ethanol and aqueous extracts. The IC50 values of DPPH assay for the H_2_O, MeOH and EtOH extracts were 111.4, 94.92 and 94.83 μg/ml; of ABTS assay were 334.3, 179.8 and 256.9 μg/ml; of H_2_O_2_ assay were 141.6, 156 and 180.6 μg/ml, respectively. The maximum radical scavenging activity was obtained in DPPH assay while the lowest scavenging activity was obtained in ABTS assay method. The data obtained in the in vitro models clearly suggest that methanol extract has higher antioxidant activity due to a higher presence of phenolic constituents in the extract.

**Conclusion:**

This study revealed that *V. amygdalina* leaf has a noteworthy antioxidant and free radical scavenging activity mitigating the traditional use of the plant for different aliments.

## Background

Plants are known to exist thousands a long time prior and have been an indispensably portion of conventional and innate therapeutic frameworks over the globe since ancient times [1, 2]. Numerous nations within the world, that is, two-third of the world’s populace depends on home grown medication for essential wellbeing care [3]. There are several traditional approaches of treating diseases using plants, Ayurveda and Siddha originated from India; and Unani in Greece. Thus, believes that every person has its own distinct temperament made up of combinations of four basic humors [4]. Medicinal plants and natural products has long been known to practitioners of Greco-Arab, Islamic medicine and Unani medicine as a therapeutic use in the treatment of diabetes and obesity which are strongly correlates deeper with the elevated risks of developing cardiovascular disease hypertension, stroke, and several malignancies [5]. Interestingly, many of the plants have analgesic properties, which can be used to relieve pain such as chronic kidney disease [6]. Plants are able to create an expansive number of diverse bioactive compounds [5, 7]. These bioactive compounds naturally found in plants and microorganism are phytochemicals [8]. These phytochemicals include alkaloids, flavonoids, saponins, terpenoids, steroids, glycosides, tannins, volatile oils, etc. [9]. Phytochemicals have several pharmacological roles such as antioxidant [10, 11], antiviral [12], anticancer [13], antimicrobial [14], antifungal [10] and antiparasitic [15]. Essential oils (volatile oils) are used in order to exploit physiological and psychological properties of individual response to volatile biostructures, with the aim to reduce stress and to speed up the healing processes [16, 17].

Lion's share of the maladies are basically connected to oxidative stress due to free radicals. Free radicals may be either oxygen derived reactive species (ROS) or nitrogen derived reactive species (RNS) [18]. Antioxidants are accepted to play a really vital part within the body defense system against free radicals [19]. The existence of antioxidants in plants is vital as numerous plants are utilize as a source of dietary antioxidants [20, 21]. High concentrations of phytochemicals, which may protect against free radical damage, accumulate in fruits and vegetables [22].

*V. amygdalina* is a perennial herb belonging to the Asteraceae family, the species indigenous to tropical Africa and is found wild or cultivated all over sub-Sahara Africa [23]. The plant is known by its local name *Grawa* in Amharic and bitter leaf in English [24]. The leaves of *V. amygdalina* have been used in Ethiopia for the treatment of different aliments such as stomach disorder, skin wound, diarrhea, scabies, hepatitis, ascarasis, tonsillitis, fever, mastitis, tapeworm and worms infection [25]. The leaves of this plant contains different bioactive compounds, including, saponins and alkaloids, terpenes, terpenes, steroids, coumarins, flavonoids, phenolic acids, lignans, xanthones, anthraquinones and sesquiterpenes [24, 26].

Plant phenols and flavonoids have antioxidant properties [27], acting as reducing agents, free radical terminators, metal chelators and singlet oxygen quenchers [28]. Hence, plants containing useful phytochemicals may supplement the of the human body by acting as natural antioxidants. These phytoconstituents depend on the geographical location where the plant material collected [29] and the season as well as the maturity of leaves [30]. The chemical profile and antioxidant activities of *V. amygdalina* grown in the study area is not reported yet. Therefore, the main purpose of this study is to investigate the qualitative phytochemical composition and total antioxidant activity and free-radical scavenging activity of the aqueous, methanol and ethanol leaf extracts of *V. amygdalina* leaf extract grows in the study area.

## Material and method

### Description of the study area

Tehuledere is one of the districts in South Wollo Zone of Amhara Regional State, which is about 430 km North of Addis Ababa, Ethiopia. It has the latitude and longitude of 11° 29′ 59.99′′N and 39°34′ 59.99′′E with an elevation between 1900–2400 m above sea level.

### Sample collection and authentication

Fresh leaves of *V. amygdalina* were collected from the area where they grow in Tehuledere district in October 2021 following the guidelines proposed by Wondafrash (2008) [31]. The plant was collected after getting written consent from the local authority and a special letter from Wollo University Postgraduate Office. The specimen was submitted to Wollo University Herbarium Center for identification and voucher number. The specimen was identified by Mr Belay Melese (Botanist) and assigned voucher number 160/2021.

### Chemicals and reagents

The analytical grade chemicals and reagents used for this study were distilled water and deionized water (H_2_O, methanol (MeOH), ethanol (EtOH), 10% ferric chloride (FeCl_3_), Wagner’s reagent (Iodine in potassium iodide), aluminum chloride (AlCl_3_), sodium nitrite (NaNO_2)_, hydrochloric acid (HCl), sulfuric acid (H_2_SO_4_), sodium hydroxide (NaOH), nitric acid (HNO_3_), sodium carbonate, iodine, NaH_2_PO_4_, Na_2_HPO_4_, DPPH, ammonium molybdate, potassium persulfate, ferric tripyridyltriazine, acetic anhydride, ascorbic acid, Fehling’s solution, etc. were used.

### Instruments and apparatus

The necessary apparatus and instruments used for this study were electronic beam balance with ± 0.0001 g precision for mass measurement, pipettes and micropipettes for measuring different amounts of acids and standard solutions, vacuum rotary evaporator for concentrating the filtrate to dryness by removing residual solvent, volumetric flasks are to be used to dilute sample solutions and prepare standard solutions. UV- visible spectrophotometer was used to measure absorbance. Soxhlet apparatus were used for extraction and electrical shaker to mix the mixture well. Digital pH meter for pH measurement, volumetric flask, beaker, conical flask with different size Beakers (50 mL, 100 mL 150 mL, 200 mL 1000 mL), Whiteman No.1 filtrate paper, separatory funnel and others were used for different purposes.

### Extraction of plant samples

The powdered plant sample was successively extracted with methanol, ethanol and aqueous solvents using Soxhlet apparatus. Three hundred gram of powdered samples of *V*. *amygdalina* leaf was placed in a thimble of Soxhlet apparatus fitted with a round bottom flask containing the desired solvent. The solvent was heated at its boiling temperature for 6 h. The solvent vapors moved upward to the condenser. The condenser changed the vapor into liquid state, and then flood into the thimble chamber until the thimble became full of solvent and sample to undergo extraction. Then, the solvent with extracted phytochemicals moved down to the round bottom flask. Round bottomed flask containing extract solution was dismantled, filtered, and the residual solvent from each extract was removed using rotary evaporator under reduced pressure. The resulting semidried mass of each fractions were stored in 4 °C refrigerator desiccators until used for experiments.

### Preliminary qualitative phytochemical screening

#### Test for alkaloids

Extracts were dissolved individually in dilute hydrochloric acid and filtered. Then the filtrates treated with Wagner s reagent (1.27 g of iodine and 2 g of KI along with 100 mL of distilled water). Formation of brown (reddish brown) precipitate indicates the presence of alkaloids [32].

#### Test for flavonoids

0.05 g of the extract was treated with few drops of 10% (w/v) sodium hydroxide solution and a few drops of concentrated H_2_SO_4_. There was no formation of yellow color indicates the absence of flavonoid [33].

#### Test for phenols

0.05 g of the extract was treated with few drops of 5% (w/v) ferric chloride solution. Formation of bluish black (blue or green) color indicates the presence of phenol [34].

#### Test for saponins

0.05 g of the extract was diluted with 20 mL of distilled water vigorously shaken in a graduated cylinder for 15 min. Formation of 1 cm layer of foam indicates the presence of saponins [35].

#### Test for glycosides

0.05 g of powdered extract was diluted with 5 mL water followed by the addition of 2 mL of glacial acetic acid and a drop of ferric chloride solution. To this, 1 mL of concentrated sulphuric acid was added very slowly. The appearance of a brown ring at the interface shows the presence of glycosides [36].

#### Test for tannins

5 g of extract was mixed into with 10 mL of distilled water. The mixture will be boiled for 5 min. The production of greenish precipitate up on the addition of 2 drops of 5% FeCl_3_ shows the presence of Tannins [37].

#### Test for steroids

50 mg of the extract was dissolved in 1 mL of chloroform. Sulphuric acid were carefully added to form a lower layer. A reddish brown color at the interface shows the presence of steroidal ring [38].

#### Test for terpenoids

2 mL of chloroform was added to plant ‘extracts (0.5 g) in a test tube. Then 3 mL of concentrated sulfuric acid was added to this mixture that result in reddish brown interface confirming the presence of terpenoids [39].

#### Test for sugars

1 mL of water and 5–8 drops of Fehling’s solution. was added to a 0.5 g of the sample and heated over water bath. The formation of brick red precipitate indicates the presence of reducing sugars [40].

### Antioxidant scavenging assay

The antioxidant activity of the aqueous, methanol and ethanol leaf extracts of *V. amygdalina* were evaluated using DPPH, ABTS, FRAP, HPOS and TAC assay methods.

### Determination of antioxidant activity of *V. amygdalina* leaves by DPPH assay

Free radical scavenging activity of different leaves extracts of *V. amygdalina* plant were measured by 1, 1- diphenyl-2-picryl hydrazyl (DPPH) [41]. Briefly, 0.1 mM solution of DPPH was prepared by dissolving 0.004 g of DPPH crystalline solid in 100 mL of analytical grade methanol and stored at 4 °C. A 4 mg of the plant extract was dissolved in 10 mL of methanol in order to prepare 400 μg/mL stock solutions and then serial dilution with methanol was performed to prepare the required concentrated solutions (50, 100,150, 200, 250, 300 μg/mL). A 2 mL of plant extract solution from each concentration was taken in a test tube and then, 3 mL of DPPH solution was added in each test tube. After 30 min incubation in the dark, the absorbance at 517 nm was recorded using a UV–Vis Spectrophotometer.

Reference standard compound being used was ascorbic acid. A stock solution of 800 μg/mL was prepared by dissolving 2 mg ascorbic acid in 2.5 mL of distilled water. Then, serial dilution with different concentrated solution was prepared (50, 100,150, 200, 250, 300 μg/mL). MeOH, EtOH, and distilled H_2_O were used as the blank for respective extracts. A mixture of 3 mL of 0.1 mM DPPH and (100 μL MeOH for methanol extract, 100 μL EtOH for ethanol extract and 100 μL H_2_O for aqueous extract) was used as control. All determinations were performed in triplicate. The percent of inhibition were plotted against concentration from which IC_50_ values were calculated.

$$\mathrm D\mathrm P\mathrm P\mathrm H\;\%\;\mathrm I\mathrm n\mathrm h\mathrm i\mathrm b\mathrm i\mathrm t\mathrm i\mathrm o\mathrm n=\frac{{\mathrm A}_{\mathrm{control}}-{\mathrm A}_{\mathrm{sample}}}{{\mathrm A}_{\mathrm{control}}}\times100$$

where, A_control_ is the mixture of methanol/ethanol/ water and DPPH solution, and A_sample_ is the mixture of sample extract and DPPH solution.

### Determination of antioxidant activity of *V. amygdalina* leaves by ABTS assay

ABTS assay was carried out using the procedure used in the previous study. ABTS radical cation was prepared by mixing 7 mM ABTS stock solution with 2.45 mM potassium persulfate in equal quantities [42]. Briefly, 7 mM ABTS solution was prepared by dissolving 0.360 g of ABTS salt in 100 mL of distilled water [43]. A 2.45 mM potassium persulfate was prepared by dissolving 0.066 g of salt in 100 mL of distilled water. Then, ABTS cation radical solution was prepared by gently mixing 10 mL of 7 mM ABTS solution and 10 mL of 2.45 mM of potassium persulfate solution, the mixture was left in dark at room temperature for 12 h until the reaction was completed and the absorbance was stabled.

The radical cation formed is further diluted in ratio (1:1) with ethanol to adjust the absorbance value to 0.700 at 734 nm using UV–Vis Spectrophotometer. A 5 μL of *V. amygdalina* leaves extract at concentrations (50, 100,150, 200, 250, 300 μg/mL) was mixed with 4000 μL of ABTS^+^• solution and allowed to stand in the dark for 2 h at room temperature. The absorbance was determined at 734 nm using a UV–Vis Spectrophotometer. Methanol, ethanol, and water were used as the blank for methanol, ethanol and aqueous extracts, respectively. A Mixtures of 10 mL of (7 mM ABTS, 2.45 mM K_2_S_2_O_8_) and (20 mL of methanol for methanol extract, 20 mL of ethanol for ethanol extract and 20 mL of water for aqueous extract) was used as control.

The reactivity of the various concentrations of each solvent extract was compared to that of ascorbic acid. All the measurements were carried out at least three times. Percent scavenging of ABTS + radical was calculated for different concentrations (50 to 300 μg/mL) of extract and standard using the following equation:

$$\mathrm A\mathrm B\mathrm T\mathrm S\;\%\;\mathrm S\mathrm c\mathrm a\mathrm v\mathrm e\mathrm n\mathrm g\mathrm i\mathrm n\mathrm g=\frac{{\mathrm A}_{\mathrm{control}}-{\mathrm A}_{\mathrm{sample}}}{{\mathrm A}_{\mathrm{control}}}\times100$$

where, A_control_ is absorbance of a mixtures of 10 mL of (7 mM ABTS, 2.45 mM K_2_S_2_O_8_) with blank solvents and A_sample_ is absorbance of the mixture of sample extract/standard and ABTS.

The antioxidant activity of *V. amygdalina* leaf extract against ABTS + • was expressed as IC50.

### Determination of total antioxidant capacity (TAC) of ***V. amygdalina*** leaves by the phosphomolybdenum Assay

The total antioxidant capacity of crude extracts was evaluated by the phosphomolybdenum assay [44]. The assay is based on the reduction of Mo(VI) to Mo(V) by the extract and subsequent formation of green phosphate (Mo(V)) complex at acidic pH [45]. One milliliter each of 0.6 M sulfuric acid, 28 mM sodium phosphate and 4 mM ammonium molybdate were added in 20 mL of distilled water and made up volume to 50 mL by adding distilled water. 0.3 mL of crude extracts of *V. amygdalina* in different concentration ranging from 50 μL to 300 μL were added to different test tubes individually containing 3 mL of reagent solution. These tubes were kept incubated at 95 °C for 90 min. Then, the absorbance of the solution was measured at 695 nm using a UV–VIS spectrophotometer against blank after cooling to room temperature. MeOH, EtOH, and H_2_O were used as the blank for respective extracts. 3 mL of a mixture of molybdate in 20 mL of water and 0.3 mL of controls were used. Ascorbic acid was used as positive reference standard. Mean values from three trials were calculated for each extract. The antioxidant capacity was estimated using the following formula:

$$\mathrm A\mathrm n\mathrm t\mathrm i\mathrm o\mathrm x\mathrm i\mathrm d\mathrm a\mathrm n\mathrm t\;\mathrm e\mathrm f\mathrm f\mathrm e\mathrm c\mathrm t\;\%=\frac{{\mathrm A}_{\mathrm{sample}}-{\mathrm A}_{\mathrm{control}}}{{\mathrm A}_{\mathrm{sample}}}\times100$$

where, A_sample_ is the absorbance of the sample and A_control_ is the absorbance of the control.

The concentration of extract at which 50% inhibition is observed (IC50) were calculated in μg/ml.

### Determination of antioxidant activity of ***V. amygdalina*** leave extracts by hydrogen peroxide scavenging (H_2_O_2_) assay

The ability of the extracts to scavenge hydrogen peroxide was determined according to the method of Al-Amiery et al. [46] with a minor modification [47, 48]. A solution of hydrogen peroxide (2 mM) was prepared in 0.2 M phosphate buffer (pH 7.4). Briefly, about 0.2 M potassium dihydrogen phosphate and 0.2 M sodium hydroxide solutions were prepared. 50 mL potassium dihydrogen phosphate solution was placed in a 200 mL volumetric flask and 39.1 mL of 0.2 M sodium hydroxide solution was added, and finally, volume was made up to 200 mL with distilled water to prepare phosphate buffer (pH-7.4). 50 mL of phosphate buffer solution was added to equal amount of hydrogen peroxide to generate the free radicals and solution was kept at room temperature for 5 min to complete the reaction.

100 μL of different concentrations (50, 100,150, 200, 250, 300 μg/mL) in distilled water of each solvent extracts were added to 600 μL of hydrogen peroxide solution. After 30 min incubation in the dark, the absorbance at 230 nm was recorded using a UV–Vis Spectrophotometer. A reference stock solution of 800 μg/mL was prepared by dissolving 2 mg ascorbic acid in 2.5 mL of distilled water. Then, serial dilution with different concentrated solution was prepared (50, 100,150, 200, 250, 300 μg/mL). MeOH, EtOH and H_2_O were used as the blank for respective extracts. All determinations were performed in triplicate. The percent of inhibition were plotted against concentration from which IC_50_ values were calculated.

$$\mathrm H2\mathrm O2\;\%\;\mathrm S\mathrm c\mathrm a\mathrm v\mathrm e\mathrm n\mathrm g\mathrm i\mathrm n\mathrm g=\frac{{\mathrm A}_{\mathrm{blank}}-{\mathrm A}_{\mathrm{sample}}}{{\mathrm A}_{\mathrm{blank}}}\times100$$

where, A_blank_ is the absorbance of the blank and A_sample_ is absorbance the sample or standards*.*

## Results

The mass of the crude extracts obtained from 400 g leaves of *V. amygdalina* using methanol, ethanol and aqueous were 76, 54 and 28 g, respectively. The fact that methanol extracts most of the constituents in the plan material as shown in Table 1.

**Table 1:** Percentage yields of the crude extracts

Solvent	Weight of crude sample	Percentage yield
MeOH extract	76 g	19.0%
EtOH extract	54 g	13.5%
H_2_O extract	28 g	7.0%
Total yield	158	39.5%

### Phytochemical analysis

This study was conducted to determine the antioxidant activity of the MeOH, EtOH and H_2_O leaf extracts of *V. amygdalina.* A preliminary qualitative phytochemical investigation was conducted to distinguish the existence or nonexistence of secondary metabolites in each solvent leaf extracts. Phytochemical investigation of the plant revealed that the three solvent extracts contained numerous bioactive compounds namely alkaloids, tannins, saponins, phenols, terpenoids, steroids, glycosides and sugars (Table 2). Because of the polarity of solvents and progressive extraction strategy, the phytochemicals recognized were more or less the same.

**Table 2:** Phytochemical Analysis of *V. amygdalina* leaf Extracts

Tests	MeOH extract	EtOH extract	H_2_O extract
Alkaloids	+	+	+
Flavonoids	-	-	-
Phenols	+	+	+
Tanins	+	+	+
Saponins	+	+	+
Terpenoids	+	+	+
Steroids	+	+	-
Sugars	-	-	+
Glycosids	+	-	+

Antioxidant and free-radical scavenging activities crude leave extracts.

### Antioxidant activity extracts by DPPH assay

The antioxidant activity of leaf extracts has been studied by its ability to reduce DPPH. Interaction of antioxidant compounds with DPPH is based on the transfer of hydrogen atom or electron to DPPH radical and converts it to 1, 1- diphenyl-2- picrylhydrazine [49, 50]. The result of reduction DPPH radicals causes discoloration from purple color to yellow pale color which demonstrates the scavenging activity [51]. The antioxidant activity of the three solvents extracts and ascorbic acid against DPPH assay was tested with concentrations ranging from 50 to 300 μg/ ml as the results shown in Table 3.

**Table 3:** DPPH scavenging activity of extracts

Conc.μg/ml	H_2_O extract			MeOH extract			EtOH extract			AA		
	A	%	IC50	A	%	IC50	A	%	IC50	A	%	IC50
**50**	0.701	60.73	111.43	0.151	91.54	94.92	0.160	91.04	94.83	0.097	94.57	91.91
**100**	0.558	68.74		0.149	91.65		0.153	91.43		0.092	94.85	
**150**	0.398	77.7		0.143	91.99		0.142	92.04		0.087	95.13	
**200**	0.288	83.87		0.138	92.27		0.129	92.77		0.084	95.29	
**250**	0.203	88.63		0.131	92.66		0.126	92.94		0.079	95.57	
**300**	0.152	91.48		0.125	93		0.118	93.39		0.075	95.8	

As shown in Table 3, both the MeOH and EtOH extracts were displayed a comparable and significant concentration-dependent free radical scavenging activity from 91.54% to 93% and 91.04% to 93.39%, respectively, compared with that of the standard AA 94.57% to 95.8%). The H_2_O leaf extract exhibited moderate activity from 60.73% to 91.48%. The IC50 values of DPPH assay for the H_2_O, MeOH and EtOH extracts were 111.4 μg/ml, 94.92 μg/ml and 94.83 μg/ml, respectively. While the standard antioxidant had an IC50 of 127.737 μg/ml. Low IC_50_ values correspond to high antioxidant activity [52]. Thus, the EtOH extract had the highest antioxidant activity among the other leaves extract which has the smallest IC50 value at higher concentration.

### Antioxidant activity of extracts by ABTS assay

The oxidation of ABTS with potassium persulfate generates ABTS radical cation [53]. This radical cation gets reduced in the presence of hydrogen donating antioxidants [54]. During this reaction, the blue ABTS radical cation was decolorized [55]. The ABTS scavenging activity of the three extracts and the absorbance were given in Table 4.

**Table 4:** Free-radical scavenging activities of extracts in ABTS assay

Conc. μg/ml	H_2_O extract			MeOH extract			EtOH extract			AA		
	A	%	IC50	A	%	IC50	A	%	IC50	A	%	IC50
**50**	0.465	12.48	334.93	0.388	26.97	179.8	0.423	20.38	256.9	0.297	44.1	127.7
**100**	0.443	16.62		0.323	39.21		0.401	24.52		0.242	54.45	
**150**	0.387	27.16		0.274	48.43		0.344	35.25		0.183	65.56	
**200**	0.374	29.61		0.233	56.15		0.331	37.7		0.142	73.27	
**250**	0.347	34.69		0.216	59.35		0.304	42.78		0.085	84	
**300**	0.339	36.19		0.202	61.98		0.299	43.72		0.051	90.4	

As the above table depicts that, as the concentration of the extract increases, the percent of inhibition also increases. The relative antioxidant activity extracts to scavenge the radical ABTS^+^ has been compared with the standard ascorbic acid. I all extracts the maximum antioxidant scavenging activity was attained at concentration of 300 μg/mL. The H_2_O and EtOH extracts showed a very weak ABTS^+^ radical scavenging activity, compared with MeOH extract. The MeOH extracts were able to scavenge 61.98% of the ABTS^+^ radical and that of the standard ascorbic acid AA 90.4%. The IC50 values of ABTS assay for the H_2_O, MeOH and EtOH were 334.3, 179.8 and 256.9 μg/ml, respectively, while the standard antioxidant had an IC50 of 127.7 μg/ml.

### Antioxidant activity of extracts by Phosphomolybdenum (TAC) assay

Phosphomolybdate assay measures the capacity of an extract to destroy a free radical by transferring an electron [56, 57]. The total antioxidant capacity (TCA) of leaf extracts of *V. amygdalina* was measured based on the reduction of molybdate (VI) to molybdate (V) [45]. The antioxidant activity of each solvent extracts at various concentrations were given in the Table 5. The TCA of the three solvent extracts compared with the reference standard with the total antioxidant value 91.35%. TAC was found to be higher in MeOH extract (77.71%) followed by EtOH extract (68.29%) at a concentration of 300 μg/ml.

**Table 5:** Total antioxidant capacity (TCA) extracts by Phosphomolybdate assay

Conc. μg/ml	H_2_O extract			MeOH extract			EtOH extract			AA		
	A	%	IC50	A	%	IC50	A	%	IC50	A	%	IC50
**50**	0.044	11.36	250.8	0.073	46.58	133.3	0.048	18.75	176.53	0.247	84.21	98.96
**100**	0.054	27.78		0.095	58.95		0.059	33.9		0.297	86.87	
**150**	0.062	37.1		0.121	67.77		0.076	48.68		0.331	88.22	
**200**	0.065	40		0.127	69.29		0.101	61.39		0.368	89.40	
**250**	0.072	45.83		0.148	73.65		0.116	66.38		0.409	90.46	
**300**	0.074	47.3		0.175	77.71		0.13	68.29		0.451	91.35	

The IC50 value of MeOH extract was 133.3 μg/ml, EtOH extract was 176.5 μg/ml, and H_2_O extract was 250.8 μg/ml. AA was used as a reference standard with IC50 value of 98.96 μg/ml. The MeOH extracts have the IC50 value closer to the standard which corresponds to high antioxidant activity.

### Hydrogen peroxide scavenging assay of extracts

The scavenging effect of different extracts of *V. amygdalina* on hydrogen peroxide was concentration-dependent (25–300 μg/mL) as shown in Table 6. The methanol extract displayed strong H_2_O_2_ scavenging activity (IC_50_ 141.6 µg/mL), whereas water extract exhibited IC_50_ value 180.6 µg/mL.

**Table 6:** Hydrogen peroxide scavenging assay of extracts

Conc. μg/ml	H_2_O extract			MeOH extract			EtOH extract			AA		
	A	%	IC50	A	%	IC50	A	%	IC50	A	%	IC50
**50**	0.453	27.87	180.6	0.322	48.73	141.6	0.399	36.46	156	0.231	63.22	112
**100**	0.359	42.83		0.287	54.3		0.308	50.96		0.199	68.31	
**150**	0.338	46.18		0.241	61.62		0.298	52.55		0.152	75.8	
**200**	0.305	51.43		0.205	67.36		0.244	61.15		0.117	81.37	
**250**	0.268	57.32		0.198	68.47		0.207	67.04		0.079	87.42	
**300**	0.219	65.13		0.186	70.38		0.198	68.47		0.047	92.52	

All solvent extracts of tested plant exhibited concentration-dependent free radical scavenging activity. The MeOH extract showed good scavenging ability (70.38%). The antioxidant activity of EtOH and H_2_O extracts were 68.47% and 65.13% respectively at a concentration of 300 μg/l. The IC50 values of MeOH, EtOH, H_2_O extracts and standard AA extract were 141.6, 156, 180.6 and 112 μg/ml, respectively. The MeOH extract had higher antioxidant activity while the H_2_O extract had the lowest antioxidant activity. The overall comparison of the antioxidant activity of the three leaf extracts of *V. amygdalina* and the standard ascorbic acid in terms of IC50 (μg /ml) and scavenging activity at 300 μg /ml were given in Table 7.

**Table 7:** Free-radical scavenging activity at 300 μg/ml and IC50 Values in μg/ml

Samples	**Antioxidant Assay**							
	**DPPH**		**ABTS**		**H**_**2**_**O**_**2**_		**TAC**	
	%I	IC50	%I	IC50	%I	IC50	%I	IC50
H_2_O extract	91.48	111.43	36.19	334.93	65.13	250.75	47.3	180.6
MeOH extract	93	94.92	61.98	179.75	70.38	133.27	77.71	141.6
EtOH extract	93.39	94.83	43.72	256.89	68.47	176.53	68.29	156
AA	95.8	91.91	90.4	127.74	92.52	98.96	91.35	112

## Discussion

**Fig. 1:**
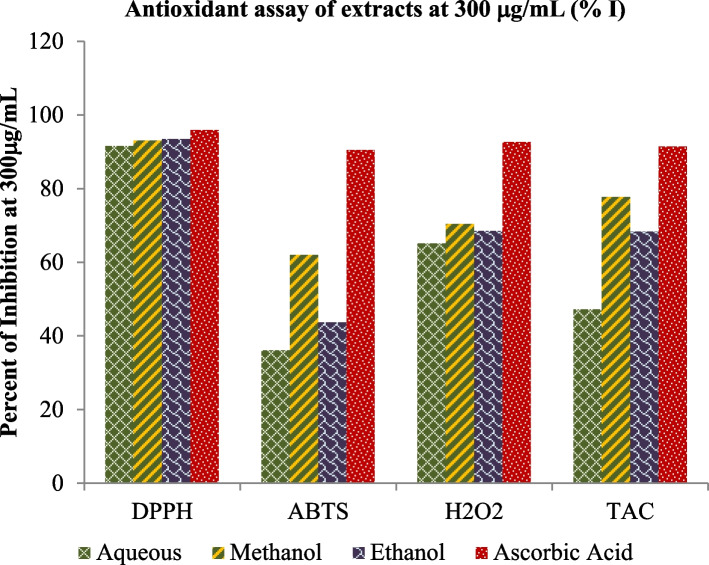
Scavenging activities of all antioxidant assays

**Fig. 2:**
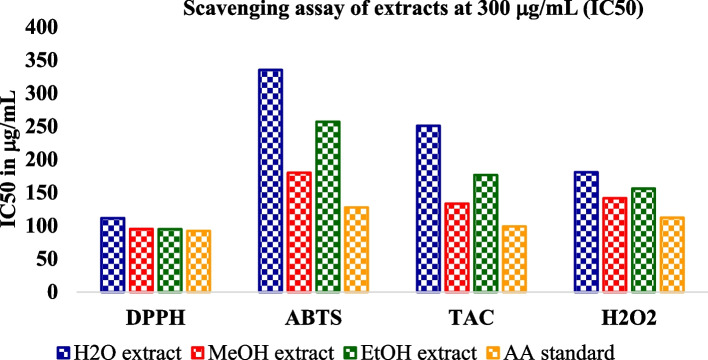
IC50 values of all antioxidant assays

The crude leaf extract contains phenolic and tannin compounds which are potential sources of antioxidants [58–60]. These bioactive compounds possess many biological properties including antioxidants such as anti-fungi [10, 11], antibacterial [11, 14], anti-inflammatory [12], antioxidant [12, 61], anticancer [12], antiviral [13]. The below figures (Figs. 1 and 2) illustrates that the antioxidant scavenging activities of all solvent extraction in DPPH assay were found to be the highest as compared to the others assays at 300 µg/mL.

The antioxidant scavenging activities of the H_2_O, MeOH and EtOH extracts and AA were 91.48%, 93%, 93.39% and 95.8%, respectively (Table 7). ABTS assay method exhibited the lowest scavenging activity in all solvent extractions at 300 µg/mL.

**Fig. 3:**
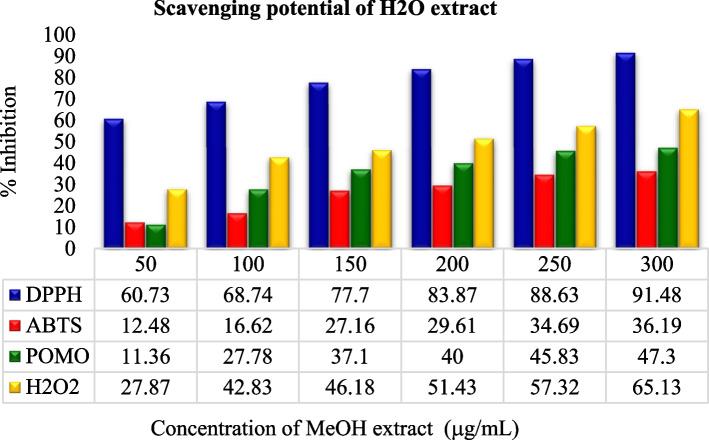
Scavenging power of aqueous (H_2_O) extract against all assays

**Fig. 4:**
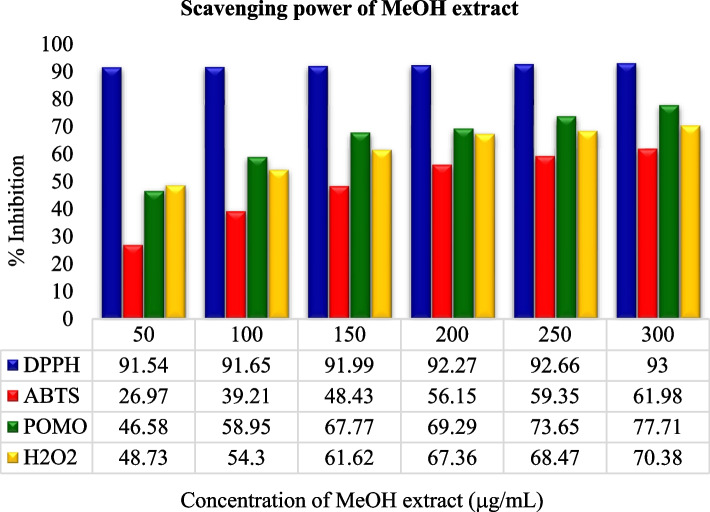
Scavenging power of methanol (MeOH) extract against all assays

**Fig. 5:**
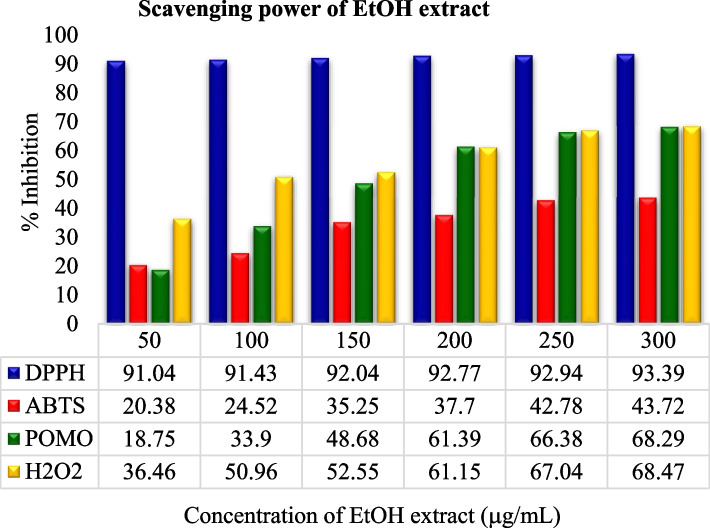
Scavenging power of ethanol (EtOH) extract against all assays

**Fig. 6:**
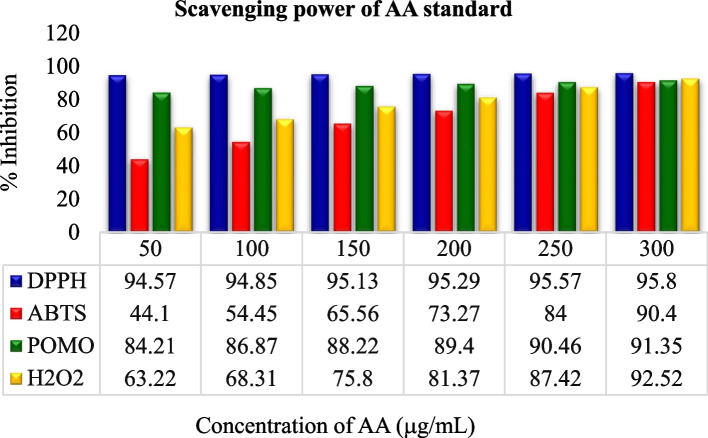
Scavenging power of standard ascorbic acid (AA) extract against all assays

In comparison, the experimental analysis of all extracts showed that for the entire examined leaf extracts rank order in terms of % inhibition and IC50 value: DPPH assay > TAC assay > H_2_O_2_ assay > ABTS assay as shown in Figs. 3, 4, 5 and 6. But it was also observed that all the sample extracts have lesser activity than that of standard AA (Fig. 6). In the various extracts the EtOH leaf showed 94.83 IC50 value in DPPH assay which is closer to the standard AA (Fig. 5). The least antioxidant activity was observed in H_2_O leaf extract which is 111.43 μg/mL in DPPH assay (Fig. 3). The ABTS assay method exhibited the least antioxidant activity in all solvent extractions with the IC50 values of 334.93 μg/mL, 256.89 μg/mL,179.75 μg/mL and 127.73 μg/mL for the H_2_O, MeOH, EtOH extracts and the standard ascorbic acid, respectively (Table 7 and Figs. 3, 4, 5 and 6). The MeOH and EtOH extracts have comparable antioxidant activity in DPPH assay with the IC50 values of 94.92 and 94.83 μg/mL, respectively (Figs. 4 and 5). However, for the remaining antioxidant assay methods the methanol extract exhibited the highest antioxidant activity and more potent than ethanol or aqueous extract.

## Conclusion

The antioxidant activity of *V. amygdalina* was evaluated by DPPH assay, ABTS assay, TAC and H_2_O_2_ assay methods. The results showed that, compared to ethanol and aqueous extracts, a methanolic leave extract has a higher percentage of inhibition of DPPH radical scavenging activity and high total antioxidant capacity. From the results of in-vitro antioxidant assays, it can be concluded that the MeOH extract of *V. amygdalina* shown concentration-dependent significant free radical scavenging activity in the order DPPH > H_2_O_2_ assay > Phosphomolybdenum assay > ABTS, and the EtOH and H_2_O extracts shown a free radical scavenging activity in the order of DPPH > Phosphomolybdenum assay > H_2_O_2_ assay > ABTS. Moreover, DPPH assay method exhibited the highest scavenging activity in all solvent extractions. The lowest antioxidant activity was obtained in ABTS scavenging assay method which is attributed to higher IC50 value in all solvent extraction. Thus, the present study suggests that MeOH extract can be used as a good source of natural antioxidants for health benefits and further isolation of bioactive compounds are required for identifying the unknown compounds to establish their pharmacological properties. We recommend the isolation and characterization of the bioactive entities and the establishment of the antioxidant mechanism of action of the extract and pure isolates.

## Data Availability

Additional data will be accessible from the corresponding author’s using sisay.awoke@wu.edu.et.

## References

[1] Süntar I (2020). Importance of ethnopharmacological studies in drug discovery: role of medicinal plants. Phytochem Rev.

[2] Fitzgerald M, Heinrich M, Booker A. Medicinal Plant Analysis: A Historical and Regional Discussion of Emergent Complex Techniques. Front Pharmacol. 2020;10:2–14. 10.3389/fphar.2019.01480 PMC6962180 31998121

[3] Oladeji OS, The Characteristics and Roles of Medicinal Plants: Some Important Medicinal Plants in Nigeria. Natural Products: An Indian Journal. 2016:12.

[4] Parveen A (2022). The traditional system of Unani medicine, its origin, evolution and Indianisation: A critical appraisal. Indian J Tradit Knowl (IJTK).

[5] Maghbool M (2020). The effects of eugenol nanoemulsion on pain caused by arteriovenous fistula cannulation in hemodialysis patients: A randomized double-blinded controlled cross-over trial. Complement Ther Med.

[6] Saad B, Kmail A, Haq SZH (2022). Anti-Diabesity Middle Eastern Medicinal Plants and Their Action Mechanisms. Evid Based Complement Alternat Med.

[7] Sytar O, Smetanska I (2022). Special Issue "Bioactive Compounds from Natural Sources (2020, 2021)". Molecules.

[8] Qin S (2010). Biodiversity, bioactive natural products and biotechnological potential of plant-associated endophytic actinobacteria. Appl Microbiol Biotechnol.

[9] Huang W (2018). Phytochemical and Pharmacological Properties of Chaenomeles speciosa: An Edible Medicinal Chinese Mugua. Evid Based Complement Alternat Med.

[10] Pinho F.V.S.d.A (2016). Phytochemical Composition, Antifungal and Antioxidant Activity of Duguetia furfuracea. Oxid Med Cell Longev.

[11] Zouirech O (2022). Phytochemical Analysis and Antioxidant, Antibacterial, and Antifungal Effects of Essential Oil of Black Caraway (Nigella sativa L.) Seeds against Drug-Resistant Clinically Pathogenic Microorganisms. Biomed Res Int.

[12] Qanash H (2022). Anticancer, antioxidant, antiviral and antimicrobial activities of Kei Apple (Dovyalis caffra) fruit. Sci Rep.

[13] Ali SK (2022). Phytochemical screening and characterization of the antioxidant, anti-proliferative and antibacterial effects of different extracts of Opuntia ficus-indica peel. J King Saud Univ Sci.

[14] Gacem MA (2019). Phytochemical screening, antifungal and antioxidant activities of three medicinal plants from Algerian steppe and Sahara (preliminary screening studies). SN Applied Sciences.

[15] Panda SK, Luyten W (2018). Antiparasitic activity in Asteraceae with special attention to ethnobotanical use by the tribes of Odisha. India Parasite.

[16] Samfira I (2015). Characterization and identity confirmation of essential oils by mid infrared absorption spectrophotometry. Dig J Nanomater Biostruct.

[17] Butu M, et al. STUDY OF ZINGIBERENE FROM LYCOPERSICON ESCULENTUM FRUIT BY MASS SPECTOMETRY. Digest Journal of Nanomaterials & Biostructures (DJNB). 2014;9(3):935–41.

[18] Phaniendra A, Jestadi DB, Periyasamy L (2015). Free radicals: properties, sources, targets, and their implication in various diseases. Indian J Clin Biochem.

[19] Lobo V (2010). Free radicals, antioxidants and functional foods: Impact on human health. Pharmacogn Rev.

[20] Kasote DM (2015). Significance of antioxidant potential of plants and its relevance to therapeutic applications. Int J Biol Sci.

[21] Carlsen MH (2010). The total antioxidant content of more than 3100 foods, beverages, spices, herbs and supplements used worldwide. Nutr J.

[22] Jideani AIO (2021). Antioxidant-rich natural fruit and vegetable products and human health. Int J Food Prop.

[23] Obarisiagbon AJ (2019). Studies on the effects of Vernonia amygdalina aqueous leaf extract on the biochemical, haematological and hypoglycemic parameters in diabetic rats: Prerequisite to formulation into pharmaceutical dosage form. J Pharm Allied Sci.

[24] Alara OR (2019). Extraction and characterization of bioactive compounds in Vernonia amygdalina leaf ethanolic extract comparing Soxhlet and microwave-assisted extraction techniques. J of Taibah Univ Sci.

[25] Swee KY (2010). Vernonia amygdalina, an ethnoveterinary and ethnomedical used green vegetable with multiple bio-activities. J Med Plants Res.

[26] Alara OR (2017). Phytochemical and pharmacological properties of Vernonia amygdalina: a review. J Chem Eng Ind Biotechnol.

[27] Igile GO (1994). Flavonoids from Vernonia amygdalina and their antioxidant activities. J Agric Food Chem.

[28] Akbarirad H, et al. An overview on some of important sources of natural antioxidants. Int Food Res J. 2016;23(3):935–41.

[29] Das K (2017). Effect of demographic location on Phlebodium decumanum (Willd.) J. Sm. for its phytoconstituents and establishment of antioxidant and novel anthelmintic activity of its aqueous and methanolic leaf extracts. Ann Phytomedicine.

[30] Ekpenyong CE, Akpan EE, Daniel NE (2014). Phytochemical constituents, therapeutic applications and toxicological profile of Cymbopogon citratus Stapf (DC) leaf extract. J Pharmacognosy Phytochemistry.

[31] Wondafrash M (2008). A preliminary guide to plant collection, identification and herbarium techniques.

[32] Farooq S (2022). Preliminary Phytochemical Analysis: In-Vitro Comparative Evaluation of Anti-arthritic and Anti-inflammatory Potential of Some Traditionally Used Medicinal Plants. Dose-Response.

[33] Anusmitha KM (2022). Phytochemical analysis, antioxidant, anti-inflammatory, anti-genotoxic, and anticancer activities of different Ocimum plant extracts prepared by ultrasound-assisted method. Physiol Mol Plant Pathol.

[34] Qiu Z, Li C-J (2020). Transformations of less-activated phenols and phenol derivatives via C-O cleavage. Chem Rev.

[35] Chen C (2022). Comparative Transcriptome and Phytochemical Analysis Provides Insight into Triterpene Saponin Biosynthesis in Seeds and Flowers of the Tea Plant (Camellia sinensis). Metabolites.

[36] Xu L-Y, Fan N-L, Hu X-G (2020). Recent development in the synthesis of C-glycosides involving glycosyl radicals. Org Biomol Chem.

[37] Chiangnoon R (2022). Phytochemical Analysis, Antioxidant, and Wound Healing Activity of Pluchea indica L.(Less) Branch Extract Nanoparticles. Molecules.

[38] Agidew MG (2022). Phytochemical analysis of some selected traditional medicinal plants in Ethiopia. Bull Natl Res Cent.

[39] Mazzara E (2022). A Comprehensive Phytochemical Analysis of Terpenes, Polyphenols and Cannabinoids, and Micromorphological Characterization of 9 Commercial Varieties of Cannabis sativa L. Plants.

[40] Nakaziba R (2022). Phytochemical Analysis, Acute Toxicity, as well as Antihyperglycemic and Antidiabetic Activities of Corchorus olitorius L Leaf Extracts. Sci World J.

[41] Oriakhi K (2013). Comparative Antioxidant Activities of Extracts of Vernonia amygdalina and Ocimum gratissimum Leaves. J Agric Sci.

[42] Gupta S (2021). Total antioxidant capacity: Relevance, methods and clinical implications. Andrologia.

[43] Noman OM (2021). Comparative study of antioxidant and anticancer activities and HPTLC quantification of rutin in white radish (Raphanus sativus L.) leaves and root extracts grown in Saudi Arabia. Open Chemistry.

[44] Anokwah D (2022). Evaluation of the anti-inflammatory and antioxidant potential of the stem bark extract and some constituents of Aidia genipiflora (DC.) dandy (rubiaceae). Heliyon.

[45] Prieto P, Pineda M, Aguilar M (1999). Spectrophotometric quantitation of antioxidant capacity through the formation of a phosphomolybdenum complex: specific application to the determination of vitamin E. Anal Biochem.

[46] Al-Amiery AA (2015). Hydrogen Peroxide Scavenging Activity of Novel Coumarins Synthesized Using Different Approaches. PLoS ONE.

[47] Hazra B, Biswas S, Mandal N (2008). Antioxidant and free radical scavenging activity of Spondias pinnata. BMC Complement Altern Med.

[48] Keser S (2012). Hydrogen Peroxide Radical Scavenging and Total Antioxidant Activity of Hawthorn. Chem J.

[49] Sreelatha S, Padma PR (2009). Antioxidant activity and total phenolic content of Moringa oleifera leaves in two stages of maturity. Plant Foods Hum Nutr.

[50] Rahman MM (2015). In vitro antioxidant and free radical scavenging activity of different parts of Tabebuia pallida growing in Bangladesh. BMC Res Notes.

[51] Akar Z, Küçük M, Doğan H (2017). A new colorimetric DPPH(•) scavenging activity method with no need for a spectrophotometer applied on synthetic and natural antioxidants and medicinal herbs. J Enzyme Inhib Med Chem.

[52] Tariq S (2022). Comparative Analysis of Antioxidants Activity of Indigenously Produced <i>Moringa Oleifera</i> Seeds Extracts. Biomed Res Int.

[53] Re R (1999). Antioxidant activity applying an improved ABTS radical cation decolorization assay. Free Radic Biol Med.

[54] Shen G-B (2021). Quantitative Estimation of the Hydrogen-Atom-Donating Ability of 4-Substituted Hantzsch Ester Radical Cations. ACS Omega.

[55] Ilyasov IR (2020). ABTS/PP Decolorization Assay of Antioxidant Capacity Reaction Pathways. Int J Mol Sci.

[56] Wan C (2011). Antioxidant activity and free radical-scavenging capacity of Gynura divaricata leaf extracts at different temperatures. Pharmacogn Mag.

[57] Mamta, et al. Antioxidants, in Biotransformation of Waste Biomass into High Value Biochemicals, S.K. Brar, G.S. Dhillon, and C.R. Soccol, Editors. 2014, Springer New York: New York, NY. p. 117–38.

[58] Muniyandi K (2019). Phenolics, tannins, flavonoids and anthocyanins contents influenced antioxidant and anticancer activities of Rubus fruits from Western Ghats. India Food Sci Hum Wellness.

[59] Olas, B., Berry Phenolic Antioxidants – Implications for Human Health? Frontiers in Pharmacology, 2018. 9. 10.3389/fphar.2018.00078 PMC5890122 29662448

[60] Foss K, Przybyłowicz KE, Sawicki T (2022). Antioxidant Activity and Profile of Phenolic Compounds in Selected Herbal Plants. Plant Foods Hum Nutr.

[61] Ahmed AO, Etonihu AC, Nweze NO (2022). Analysis of Chemical Compositions of Portland Cement and Limestone from Four Geopolitical Zones of Nigeria. J Minerals Mater Characterization Eng.

